# Psychological Predictors of Bullying in Adolescents From Pluricultural Schools: A Transnational Study in Spain and Ecuador

**DOI:** 10.3389/fpsyg.2019.01383

**Published:** 2019-06-19

**Authors:** Antonio J. Rodríguez-Hidalgo, Yisela Pantaleón, Juan Calmaestra

**Affiliations:** ^1^ Department of Psychology, Universidad de Córdoba, Córdoba, Spain; ^2^ Department of Education, University Laica Eloy Alfaro of Manabí, Manta, Ecuador

**Keywords:** bullying, ethnic-cultural discrimination, social skills, empathy, self-esteem

## Abstract

This study aimed to analyze the levels of personal aggression and victimization, ethnic-cultural aggression and victimization, self-esteem, empathy, social skills and gender in adolescents as potential predictors of bullying in Spain and Ecuador. The wide pluricultural sample comprised secondary education students from both countries (*N* = 25,190, average age = 13.92, SD = 1.306; *N*_Spain_ = 14,437; *N*_Ecuador_ = 10,753), who took part in the study by filling in a self-report. The results revealed that predictive models of bullying for both countries explain 50–70% of variance. A transnational predictive pattern of personal victimization can be observed based on the levels of ethnic-cultural victimization, ethnic-cultural aggression, personal aggression, self-deprecation, and affective empathy. A transnational predictive pattern of personal aggression is evidenced depending on the levels of ethnic-cultural aggression, personal victimization, self-deprecation, ethnic-cultural victimization, and the fact of being female. We concluded that bullying can largely be predicted by involvement in ethnic-cultural discrimination. These results are discussed, and educational inferences are drawn for prevention.

## Introduction

School is a developmental context for adolescents where they have the opportunity to interact and join a peer group (Salmivalli, 2010; Eccles and Roeser, 2011). In recent decades, the school population has been more and more culturally diverse due to globalization and the increase in migratory flows (Hull and Hellmich, 2018; Kastoryano, 2018). This makes that peer relationships are also relationships between different cultural groups (Marks et al., 2014; Rodríguez-Hidalgo et al., 2018).

In the last decades, the phenomenon of interpersonal violence known as bullying has been the major concern in schools. This phenomenon is characterized by a perverse dynamic in which the roles of bully and victim emerge (Hong and Espelage, 2012; Beltrán-Catalán et al., 2018). The victim is subdued by the bully or bullies in terms of intentional physical and/or psychological damage, repeatedly over time and in an asymmetrical power relation (Olweus, 2013). This abuse is an immoral behavior that breaks the most basic rules of peer reciprocity (Ortega-Ruiz et al., 2012) and makes the victim feel more and more vulnerable and unable to protect him/herself (Sentse et al., 2017). Bullying victimization has serious consequences. For example, at an academic level, a lower performance, the desire of not attending school or even a school dropout could be observed, whereas at a healthy level, anxiety, depression, sleep disturbance, self-harm, suicide attempts or even suicide could appear (Wolke and Lereya, 2015). Bullies and bullying bystanders also suffer the negative effects of this violent phenomenon since they take the risk of internalizing and consolidating aggressive, immoral and little empathic interrelational patterns as well as suffering social imbalances throughout their development (Vanderbilt and Augustyn, 2010; Ortega-Ruiz et al., 2012). Some studies have revealed that there is an overlap between aggression and victimization in bullying; in fact, they even show that they seem to intertwine (e.g., Mishna, 2003; Del Rey et al., 2012).

Insofar as cultural diversity is present in schools, educational centers are concerned about the ethnic-cultural discrimination that threatens and harms the health and development of students from cultural minorities (e.g., Priest et al., 2014; Cooper and Sánchez, 2016; Brüggemann and D’Arcy, 2017). Ethnic-cultural aggressions predict the psychological imbalance of those who suffer them (Benner and Graham, 2013) and act as an educational barrier (Baysu et al., 2016). The co-occurrence of bullying victimization and ethnic-cultural discriminatory victimization makes more predictable the emergence of suicidal ideation than when these phenomena occur separately (Garnett et al., 2014).

In order to prevent and palliate bullying among students, more and more researchers have stated that their predictors and associated factors should be studied in different cultural contexts and within different ethnic-cultural groups (e.g., Garnett et al., 2014; Vera et al., 2017). Several transnational studies on ways of violence in minors have shown consistency in some predictors and inconsistency in others, allowing us to propose strategies adapted to every country (e.g., Rodríguez-Hidalgo et al., 2018). Comparing transnational knowledge may help to fit prevention and intervention to every single context (e.g., Arenas et al., 2015; Mucci et al., 2016; Bartoll et al., 2019). In order to foresee the emergence and consolidation of the dynamics of bullying, it is necessary to know more about the characteristics of personality and the types of interpersonal behaviors that allow us to predict this phenomenon. Hereunder, there is a review about some of the potential bullying predictive factors in adolescents from culturally diverse schools: (1) personal, such as self-esteem, empathy, social skills and gender; (2) and interpersonal, like personal victimization and personal aggression (bullying), or ethnic-cultural victimization and ethnic-cultural aggression (discrimination).

There is a negative association between the levels of victimization and self-esteem in adolescents (Blood et al., 2011; Chen and Wei, 2011; Rodríguez-Hidalgo et al., 2015). Additionally, victimization is negatively linked to social adjustment and to the number of friends in school (Rodríguez-Hidalgo et al., 2014). Those adolescents who suffer bullying victimization take a greater risk of internalizing problems like having a low global self-esteem (Mishna et al., 2016). On the other hand, it has been observed that self-esteem acts as an internal element influencing on the overcoming of victimization experiences (Sapouna and Wolke, 2013), so it can be considered as a protective factor. It seems that there is a cause-effect relation between victimization and self-esteem (Fredstrom et al., 2011).

Tsaousis’ review and meta-analysis (2016) reveal that the existing negative association between aggression and self-esteem is weaker than the one between victimization and self-esteem (Tsaousis, 2016). This researcher underscores that the results from different studies regarding this subject support two hypotheses: low self-esteem acts as a precursor of aggression toward peers; and in adolescents with high self-esteem, they develop aggressive behaviors toward their peers when their self-esteem is threatened by them.

Part of the studies on cognitive empathy and involvement in bullying has found a link between both (e.g., Caravita et al., 2009; Dini et al., 2016; Rieffe and Camodeca, 2016), while another part has not found any relation between them (van Noorden et al., 2015). However, within the studies that have evidenced such connection, the results are inconsistent. In some studies, it is said that cognitive empathy is positively associated with a violent behavior in both genders (Caravita et al., 2009; Rieffe and Camodeca, 2016). However, other studies conclude that cognitive empathy is negatively associated with levels of aggression in bullying (Mitsopoulou and Giovazolias, 2015; Dini et al., 2016). One of the few existing longitudinal studies has revealed that cognitive empathy does not predict involvement as a bully or the other way around (Stavrinides et al., 2010). Nevertheless, there are studies stating that low cognitive empathy and gender—being male—are predictors of an aggressive behavior (Kokkinos and Kipritsi, 2012).

Affective empathy is negatively associated with aggression in bullying (Mitsopoulou and Giovazolias, 2015; van Noorden et al., 2015; Rieffe and Camodeca, 2016), especially more in boys than girls (Caravita et al., 2009). It has been seen that a low affective empathy is a predictor of the involvement as a bully and the other way around (Stavrinides et al., 2010). However, other studies do not show any association between affective empathy and involvement in bullying (Dini et al., 2016).

A negative correlation has been observed between the levels of victimization and cognitive and affective empathy (Kokkinos and Kipritsi, 2012). van Noorden et al.’s systematic review (2015) concludes that victimization is not usually associated with affective empathy, but it is usually negatively associated with cognitive empathy: the victims experience what others feel but they do not understand what they feel.

Adolescents’ assertive responses to different situations in the school context are considered as more effective by peers and teachers, so they are more adaptive (Dirks et al., 2009, 2014). Girls give more assertive responses in comparison to boys when facing challenging situations between peers (Rose and Rudolph, 2006). In adolescents, educational training in assertiveness is seen as a solution for bullying (Boket et al., 2016), since it can improve the coping of social situations, modify the aggressive behavior, improve social skills, and balance the emotional status (Keliat et al., 2015). Several recent studies have revealed that the educational intervention to promote assertive behaviors has a positive effect in: (1) the reduction of victimization (Keliat et al., 2015; Avşar and Ayaz-Alkaya, 2017), and (2) the reduction of aggressive behaviors toward peers in bullying (Ttofi and Farrington, 2011; Schroeder et al., 2012; Keliat et al., 2015; Avşar and Ayaz-Alkaya, 2017).

Despite the fact that many existing educational programs for the prevention of bullying in schools try to promote the development of conflict-resolution skills, a very little percentage of adolescents has shown to have used these skills to deal with bullying situations (Didaskalou et al., 2017). It has been seen that cooperative resolution skills negatively correlate with the emission of aggressive behaviors and the justification and acceptance of a violent behavior; however, these skills positively correlate with empathy toward bullying victims (Garaigordobil, 2012, 2017).

The high belief about their skills to communicate, resolve conflicts, and handle emotions with friends is related to low levels of victimization in adolescents (Fitzpatrick and Bussey, 2014). Bullying victims show less communicative and conflict-resolution skills (Haynie et al., 2001; Kochel et al., 2015). On the other hand, bullies show less communicative skills associated with a successful performance in collaborative tasks than defenders; bullies give less useful explanations and less guidance instructions to their peers than defenders (Murphy and Faulkner, 2011).

In the last two decades, attention has increasingly been paid in studies to the weight of cultural diversity regarding coexistence and violence phenomena among peers. Some studies reveal that the higher the presence of ethnic-cultural diversity is among students, the higher the bullying prevalence will be (Vervoort et al., 2010; Jansen et al., 2016). In those adolescents who belong to cultural minorities, the content of the suffered intimidation is often based on the differences of origin, ethnic group, language or migratory status (Maynard et al., 2016; Brietzke and Perreira, 2017). It has been observed that the minority status within each school is related both to the general or personal victimization and to the particularly racist victimization (Fisher et al., 2015). Being a victim of physical, verbal or relational bullying shows a strong association with being a victim of ethnic-cultural discrimination (Monks et al., 2008; Rodríguez-Hidalgo et al., 2014, 2015; Cardoso et al., 2017). Additionally, it has been seen that being a victim of discrimination between adolescents from different ethnic groups is positively related to being a victim of discrimination between peers from the same ethnic group (Benner and Wang, 2017). Nevertheless, there is a research gap regarding the connection between personal aggression in bullying and ethnic-cultural discriminatory aggression.

In view of the increasing study of bullying concerning ethnic-cultural diversity in the last years, some educational projects addressing discrimination among peers (Rodríguez-Hidalgo et al., 2017; Earnshaw et al., 2018) are being developed. However, it is necessary to have a greater body of knowledge about the nature of the relations between bullying and ethnic-cultural discrimination so that these educational proposals are more effective.

## The Present Study

The review of scientific literature enables us to observe that there are hardly any transnational studies about bullying predictors—personal aggression and personal victimization—that has been carried out with a wide sample. Thus, we need to know if they act as bullying predictors as well as to what extent they are stable or different among countries: (1) personal factors such as self-esteem, empathy, social skills, and gender and (2) interpersonal factors such as the levels of involvement in personal victimization (bullying), personal aggression (bullying), ethnic-cultural victimization (discrimination), and/or ethnic-cultural aggression (discrimination). We have carried out a comparative study on students from two different countries: Spain and Ecuador. These two countries have significant cultural links; however, they deal with ethnic-cultural diversity in different ways. Ecuador is multiethnic and multicultural, while Spain is neither multiethnic nor multicultural despite having recognized rights to cultural diversity. The objectives of the present study are:

1. To know what personal and interpersonal factors are predictors of personal victimization.
2. To know what personal and interpersonal factors are predictors of personal aggression.
3. To know which predictive patterns of personal victimization and personal aggression are common in Spain and Ecuador and which ones are unique in each country.

The following hypotheses have been studied:

1. Personal aggression and personal victimization will be predicted by means of social skills, self-esteem, and empathy.
2. Being female will be a positive predictor of personal victimization and a negative predictor of personal aggression.
3. Personal aggression and personal victimization will reciprocally be positive predictors.

Ethnic-cultural aggression and ethnic-cultural victimization will be predictors of personal aggression and personal victimization.

## Materials and Methods

### Participants

A total of 25,190 participants took part in the study. Two data collections were carried out following the same methodology, one in all Spain and another one in Ecuador. The Spanish sample was obtained in the whole national territory, while the Ecuadorian sample was obtained in zone 4 of Ecuador, composed of the extensive provinces of Manabí and Santo Domingo de Los Tsáchilas. The subjects were selected using a random cluster sampling. From the total sample, 42.7% (10,753 subjects) were from the Ecuadorian collection, while 57.3% (14,437 subjects) were from the Spanish collection. The participants were studying in equivalent years within the two different educational systems. The average age of the sample is 13.92 (SD = 1.306), being the average age of the Spanish sample 14.03 (SD = 1.390) and of the Ecuadorian sample 13.77 (SD = 1.169). Regarding gender, a similar proportion of boys and girls was found in the sample (49.9% were girls). For the Spanish sample, this percentage amounted to 50.7% and for the Ecuadorian sample, it fell to 48.9%.

### Instruments

To measure the levels of bullying aggression and victimization among peers, we used the European Bullying Intervention Project Questionnaire—EBIPQ—(Ortega-Ruiz et al., 2016). The questionnaire is composed of 14 items, 7 items regarding victimization and 7 for aggression. Those items have five response options (1 = never; 2 = once or twice; 3 = once or twice a month; 4 = about once a week; 5 = more than once a week). The reliability indexes are high, not only for the Spanish sample (*α*_victimization-SPA_ = 0.827; *α*_aggression-SPA_ = 0.823) but also for the Ecuadorian one (*α*_victimization-ECU_ = 0.826; *α*_aggression-ECU_ = 0.855). The CFA showed optimal levels for the two-factor structure of the instrument (*χ*^2^ S-B = 10371.7069, *p* = 0.00; CFI = 0.97; NNFI = 0.96; RMSEA = 0.067).

An adaptation of the EBIPQ was used to measure involvement in victimization and aggression due to ethnic-cultural discrimination: the European Bullying Intervention Project Questionnaire—Ethnic-Cultural Discrimination Version (EBIPQ-ECD Rodríguez-Hidalgo et al., 2019). The EBIPQ-ECD has a similar structure to the EBIPQ, presenting the same number of items and the same measurement scale. Its psychometric properties in this study are even better than the ones of the EBIPQ for both the Spanish sample (*α*_victimization-SPA_ = 0.862; *α*_aggression-SPA_ = 0.855) and the Ecuadorian sample (*α*_victimization-ECU_ = 0.858; *α*_aggression-ECU_ = 0.873). The CFA showed optimal levels for the two-factor structure of the instrument (*χ*^2^ S-B = 12087.4424, *p* = 0.00; CFI = 0.98; NNFI = 0.97; RMSEA = 0.071).

To address the measurement of self-esteem, the Rosenberg Self-Esteem Scale—RSES—(Rosenberg, 1989) was used, adapted, and validated by Martín-Albo et al. (2007) and Viejo (2014). The scale has widely been used with samples of adolescents. The two-factor model of the scale fits the best for this study, independently considering self-confidence (five items positively formulated) and self-deprecation (five items negatively formulated). The values of the scale of measurement range from 1 to 4. The reliability analyses have shown enough indexes for both the Spanish sample (*α*_self-confidence-SPA_ = 0.816; *α*_self-deprecation-SPA_ = 0.808) and the Ecuadorian sample (*α*_self-confidence-ECU_ = 0.719; *α*_self-deprecation-ECU_ = 0.645). The CFA showed optimal levels for the two-factor structure of the instrument (*χ*^2^ S-B = 2906.1713, *p* = 0.00; CFI = 0.98; NNFI = 0.97; RMSEA = 0.053).

The Basic Empathy Scale (BES; Jolliffe and Farrington, 2011) was used to measure the participants’ levels of empathy, specifically the Oliva et al.’s adaptation (2011) of nine items, distinguishing between affective empathy (four items) and cognitive empathy (five items). The scale of measurement of each item ranges from 1 (*Totally disagree*) to 5 (*Totally agree*). The reliability values for both subscales are very high not only for the Spanish sample (*α*_cognitive_empathy-SPA_ = 0.867; *α*_affective_empathy-SPA_ = 0.825) but also for the Ecuadorian sample (*α*_cognitive_empathy-ECU_ = 0.901; *α*_affective_empathy-ECU_ = 0.833). The CFA showed optimal levels for the two-factor structure of the instrument (*χ*^2^ S-B = 4087.9534, *p* = 0.00; CFI = 0.99; NNFI = 0.99; RMSEA = 0.070).

The social skills scale (Oliva et al., 2011) is composed of 12 items with values ranging from 1 (*Totally false*) to 7 (*Totally true*). The scale is divided into three sub-dimensions: Communicative or relational skills (five items), assertiveness (three items), and conflict-resolution skills (four items). The scores of the reliability tests are acceptable or good for most subscales in both samples (*α*_communicative_social-SPA_ = 0.774; *α*_assertiveness-SPA_ = 0.644; *α*_conflict-resolution-SPA_ = 0.767; *α*_communicative_social-ECU_ = 0.832; *α*_assertiveness-ECU_ = 0.779; *α*_conflict-resolution-ECU_ = 0.822). The CFA showed optimal levels for the three-factor structure of the instrument (*χ*^2^ S-B = 4704.5658, *p* = 0.00; CFI = 0.98; NNFI = 0.97; RMSEA = 0.056).

### Procedure

We proceeded to obtain the authorization from the respective educational administration and the educational centers in both countries. Written informed parental/guardian consent was obtained by means of the support of educational centers. The students were informed and ensured of the anonymous, confidential, and voluntary nature of their participation in the study. The registered data were codified in a data matrix through the SPSS 22 software. The collection was carried out in Ecuador by means of paper questionnaires, whereas it was done by online questionnaires in Spain. The procedure was approved by the Ethics committee of the University of Córdoba and was conducted following the national and international ethical standards.

### Analysis

For this study, multiple linear regressions were used to explore what the predictive variables of aggression and victimization were in traditional bullying. The other variables have been used as predictors with the “Introduce” method for that purpose. The collinearity diagnostics were optimal. The levels of tolerance ranged from 0.34 to 0.94 for all the variables considered in the study. VIF values ranged from 1.06 to 3.48 and Durbin-Watson statistic showed values between 1.797 and 1.947 in all the cases. All the variables included in the regressions were measured in scale, except the gender variable, which was conversed in a dummy variable regarding the gender “girl” (0 = otherwise and 1 = girl).

## Results

Overall, four multiple linear regressions were carried out to address the research objectives. In all the models, the following phenomena were included as independent variables: received ethnic-cultural victimization, ethnic-cultural aggression, self-confidence, self-deprecation, affective empathy, cognitive empathy, communication or relationship skills, assertiveness, conflict-resolution skills, and belonging to the “girl” category. This last variable was recoded in a dummy variable as it has already been mentioned. Similarly, victimization was included for the regression predicting aggression and the other way around.

The predictive model for victimization explained nearly 70% of the variance in the Spanish sample. Almost all the variables showed certain influence on the regression model for victimization in Spain, except cognitive empathy and gender. The strongest detected predictor is the score in ethnic-cultural victimization (*β* = 0.757; *p* = 0.000), followed by the involvement as traditional bully (*β* = 0.368; *p* = 0.000). A high score in ethnic-cultural aggression was found as a remarkable protective factor of this type of victimization (*β* = −0.298; *p* = 0.000). As remarkable psychological variables, high levels of self-deprecation predict a higher score in victimization (*β* = 0.078; *p* = 0.000), unlike self-confidence (*β* = −0.043; *p* = 0.000). See Table 1 for the details of parameter estimates in the analyses.

**Table 1:** Multiple linear regression model for personal victimization (Spain).

∆*R* ^2^	Model	Unstandardized coefficients		Standardized coefficients	*t*	*p*
		*B*	Std. error	*β*		
0.696	(Constant)	1.368	0.183		7.488	0.000
	Personal aggression	0.482	0.010	0.368	46.734	0.000
	Ethnic-cultural victimization	0.841	0.006	0.757	138.156	0.000
	Ethnic-cultural aggression	−0.437	0.012	−0.298	−36.543	0.000
	Self-confidence	−0.055	0.007	−0.043	−7.747	0.000
	Self-deprecation	0.088	0.006	0.078	14.034	0.000
	Affective empathy	0.027	0.006	0.026	4.188	0.000
	Cognitive empathy	0.009	0.007	0.009	1.364	0.173
	Communication/relationship skills	−0.009	0.003	−0.015	−2.980	0.003
	Assertiveness	0.022	0.007	0.020	3.092	0.002
	Conflict resolution skills	−0.010	0.005	−0.013	−1.973	0.049
	Girl (1 = yes)	0.006	0.044	0.001	0.145	0.885

Regarding the Ecuadorian sample, the predictive model explained over 50% of variance. In such model, the score in victimization was detected to be predicted by means of a high score in ethnic-cultural victimization (*β* = 0.535; *p* = 0.000) and a high score in traditional aggression (*β* = 0.382; *p* = 0.000). However, high scores in ethnic-cultural aggression predicted low levels of traditional victimization (*β* = −0.135; *p* = 0.000). As remarkable psychological variables, self-deprecation (*β* = 0.035; *p* = 0.000), cognitive empathy (*β* = 0.037; *p* = 0.002), and affective empathy (*β* = 0.039; *p* = 0.000) could be mentioned. The variables that in this case did not significantly predict the model were self-confidence, being girl and all the variables related to social skills. See Table 2 for the details of parameter estimates in the analyses for the Ecuadorian sample.

**Table 2:** Multiple linear regression model for personal victimization (Ecuador).

∆*R* ^2^	Model	Unstandardized coefficients		Standardized coefficients	*t*	*p*
		*B*	Std. error	*β*		
0.507	(Constant)	1.485	0.226		6.560	0.000
	Personal aggression	0.438	0.011	0.382	41.140	0.000
	Ethnic-cultural victimization	0.580	0.010	0.535	60.462	0.000
	Ethnic-cultural aggression	−0.160	0.012	−0.135	−13.223	0.000
	Self-confidence	−0.006	0.011	−0.004	−0.528	0.597
	Self-deprecation	0.054	0.011	0.035	4.982	0.000
	Affective empathy	0.041	0.012	0.039	3.540	0.000
	Cognitive empathy	0.030	0.009	0.037	3.169	0.002
	Communication/relationship skills	0.004	0.006	0.007	0.705	0.481
	Assertiveness	−0.006	0.012	−0.007	−0.538	0.590
	Conflict resolution skills	−0.016	0.009	−0.021	−1.821	0.069
	Girl (1 = yes)	0.047	0.072	0.005	0.659	0.510

Regarding aggression, the model predicted over 70% of variance in the Spanish sample. Only three variables did not make a significant contribution to the model: affective empathy, cognitive empathy, and conflict-resolution skills. The variable with the highest explanatory force of traditional aggression was ethnic-cultural aggression (*β* = 0.784; *p* = 0.000), followed by traditional victimization (*β* = 0.358; *p* = 0.000). As a negative predictor, ethnic-cultural victimization is emphasized (*β* = −0.240; *p* = 0.000). The higher the level of ethnic-cultural victimization is, the lower the level of traditional aggression will be. The psychological variable with the highest influence on the model was communicative skills (*β* = 0.032; *p* = 0.000), predicting high levels of traditional aggression. Being a girl was a protection factor (*β* = −0.036; *p* = 0.000) in comparison to traditional aggression. See Table 3 for the details of parameter estimates in the analyses.

**Table 3:** Multiple linear regression model for personal aggression (Spain).

∆*R* ^2^	Model	Unstandardized coefficients		Standardized coefficients	*t*	*p*
		*B*	Std. error	*β*		
0.704	(Constant)	0.577	0.138		4.196	0.000
	Personal victimization	0.273	0.006	0.358	46.734	0.000
	Ethnic-cultural victimization	−0.203	0.007	−0.240	−30.038	0.000
	Ethnic-cultural aggression	0.876	0.006	0.784	147.845	0.000
	Self-confidence	0.015	0.005	0.015	2.807	0.005
	Self-deprecation	0.013	0.005	0.015	2.740	0.006
	Affective empathy	−0.003	0.005	−0.004	−0.691	0.489
	Cognitive empathy	0.002	0.005	0.002	0.310	0.757
	Communication/relationship skills	0.014	0.002	0.032	6.432	0.000
	Assertiveness	−0.013	0.005	−0.015	−2.410	0.016
	Conflict resolution skills	−0.004	0.004	−0.007	−1.152	0.249
	Girl (1 = yes)	−0.240	0.033	−0.036	−7.318	0.000

Regarding the sample collected in Ecuador, the model of traditional aggression explained 54% of variance. In this regression, self-confidence, cognitive empathy, and the three variables related to social skills did not provide a significant effect to the model. The predictor with the greatest proportion in traditional aggression was aggression due to ethnic-cultural reasons (*β* = 0.535; *p* = 0.000), followed by the score in traditional victimization (*β* = 0.356; *p* = 0.000). The higher the scores of ethnic-cultural victimization are, the lower the levels of traditional aggression are (*β* = −0.064; *p* = 0.000). The most important psychological variables in the model were self-deprecation as a positive factor (*β* = 0.036; *p* = 0.000) and cognitive empathy as a negative factor (*β* = −0.034; *p* = 0.001). Being a girl proved to be a factor negatively related to the score in traditional aggression (*β* = −0.033; *p* = 0.000). See Table 4 for the details of parameter estimates in the analyses.

**Table 4:** Multiple linear regression model for personal aggression (Ecuador).

∆*R* ^2^	Model	Unstandardized coefficients		Standardized coefficients	*t*	*p*
		*B*	Std. error	*β*		
0.540	(Constant)	2.051	0.190		10.798	0.000
	Personal victimization	0.311	0.008	0.356	41.140	0.000
	Ethnic-cultural victimization	−0.060	0.009	−0.064	−6.476	0.000
	Ethnic-cultural aggression	0.553	0.009	0.535	62.797	0.000
	Self-confidence	−0.011	0.009	−0.009	−1.237	0.216
	Self-deprecation	0.049	0.009	0.036	5.338	0.000
	Affective empathy	−0.032	0.010	−0.034	−3.242	0.001
	Cognitive empathy	−0.004	0.008	−0.006	−0.513	0.608
	Communication/relationship skills	0.000	0.005	0.000	0.046	0.964
	Assertiveness	−0.018	0.010	−0.023	−1.869	0.062
	Conflict resolution skills	0.013	0.007	0.020	1.795	0.073
	Girl (1 = Yes)	−0.296	0.061	−0.033	−4.894	0.000

## Discussion and Conclusions

The predictive models provided for personal victimization and personal aggression (bullying) explain almost three-quarters of the variance in the Spanish sample and half of the variance in the Ecuadorian sample. Within the introduced variables, interpersonal variables showed a greater predictive weight on peer victimization and aggression than personal variables. The weight and presence of predictor factors, not only in bullying victimization but also in bullying aggression, have partially been similar in the samples collected in Spain and Ecuador. Some predictive patterns are common in both countries, but others are unique.

Regarding bullying victimization, the conclusion is that there is a fairly robust transnational predictive pattern: there are three significantly important predictors, which are victimization due to ethnic-cultural discrimination, aggression because of bullying and aggression due to ethnic-cultural discrimination, and two less important predictors that are self-deprecation and affective empathy. Ethnic-cultural victimization is a positive predictor of victimization, being the one with the greatest predictor power among all the factors that we studied. Bullying aggression is a great positive predictor of victimization, whereas ethnic-cultural aggression is also a negative predictor at a similar level. Both self-deprecation and affective empathy act as positive predictors of bullying victimization.

Beyond this homogeneous prediction pattern of bullying victimization detected in both countries, the transnational comparison enables us to emphasize some unique predictors. In the Spanish sample, we observed more predictive lightweight factors of victimization: assertiveness as a positive predictor, self-confidence, communication and relationship, and conflict-resolution as negative predictors. In contrast, we observed cognitive empathy, being a positive predictor, as a predictive lightweight factor in the Ecuadorian sample. Social skills—assertiveness, communication and relationship, conflict-resolution—play a predictor role in bullying victimization in Spain, whereas it does not play it in Ecuador.

Regarding victimization, self-esteem and empathy both have a statistically significant influence on victimization in the Ecuadorian sample; however, social skills do not have this influence. The first hypothesis of the study is therefore partially corroborated. Nevertheless, regarding the Spanish sample, the first hypothesis is corroborated, since the three personal factors that were studied make possible the prediction of victimization. The fact that social skills predict victimization is consistent with the results obtained in some studies (Haynie et al., 2001; Fitzpatrick and Bussey, 2014; Kochel et al., 2015; Jenkins and Fredrick, 2017). However, despite the fact that several studies have revealed that assertiveness acts as a protective factor of victimization (Keliat et al., 2015; Boket et al., 2016), strangely enough, in our study, it has been observed that it acts as a positive predictor of victimization. The fact that low self-esteem predicts high levels of victimization is consistent with some observations from previous studies (Blood et al., 2011; Chen and Wei, 2011; Rodríguez-Hidalgo et al., 2014, 2015). High self-esteem, as Sapouna and Wolke (2013) state, acts as a protective factor of victimization. Affective empathy was expected not to be a predictor of victimization (van Noorden et al., 2015); however, it has been evidenced that affective empathy is a positive predictor of victimization in Spain and in Ecuador. On the other hand, the expectation of cognitive empathy negatively predicting victimization was not met. Unexpectedly, cognitive empathy acts as a positive predictor of victimization in the Ecuadorian sample. These last conclusions are conflicting with the negative correlations found by Kokkinos and Kipritsi (2012) between empathy—affective and cognitive empathy—and victimization.

Regarding bullying aggression, the conclusion is that there is a stable transnational predictive pattern: ethnic-cultural aggression, bullying victimization, and self-deprecation are positive predictors, while ethnic-cultural victimization and being a girl are negative predictors. Ethnic-cultural aggression and bullying victimization are the most powerful predictors in Spain and Ecuador. Nevertheless, ethnic-cultural victimization is also a great predictor in Spain, while it is a less important predictor in Ecuador. Being a girl and self-deprecation are lightweight predictors in both countries. Regarding lightweight predictors of aggression, the conclusion is that there are specificities in the contrast by countries. Communication and relationship skills as well as self-confidence are considered positive predictors in Spain, whereas assertiveness is a negative predictor. Affective empathy is a negative predictor in Ecuador. Just like in the prediction of victimization, social skills again seem to be relevant in Spain, in contrast with the lack of predictive importance they appear to have in Ecuador.

In terms of aggression, self-esteem and empathy make possible the prediction of aggression in the Ecuadorian sample. Additionally, social skills do not contribute in a statistically significant way to the model. Considering the Spanish sample in the aggression model, both social skills and self-esteem have a statistically significant effect whereas empathy does not. This evidence about the two samples regarding aggression enables us to partially confirm the first hypothesis of the study. The observation in the Spanish sample that low assertiveness predicts aggression is aligned with the model of social-skill deficit regarding aggression (Crick and Dodge, 1994; Camodeca and Goossens, 2005); while the observation that high social and relational skills predict aggression seems to support the model of a bully with popularity and leadership while she/he manipulates his/her classmates. This model typically appears in contexts in which the norms and the educational action try to prevent and palliate bullying (Waasdorp et al., 2013; Rivas-Drake et al., 2014; Vera et al., 2017). Apart from that, the expectation of aggression being predicted by means of low levels of self-esteem was met in the Ecuadorian and Spanish samples. High self-deprecation predicted aggression in both countries; however, high self-esteem also predicted aggression only in Spain. These two opposed conclusions seem to support, respectively, two hypotheses issued by Tsaousis (2016) following his extensive review: low self-esteem acts as a precursor of aggression, while high self-esteem is a precursor of aggressive behaviors toward peers in response to the peers’ threat. As expected, the levels of cognitive empathy do not predict aggression in any of the countries of the study. It has also been evidenced that low affective empathy predicts aggressive behaviors in Ecuador. These results are consistent with the conclusions drawn by one of the few longitudinal studies carried out in that respect (Stavrinides et al., 2010). On the other hand, these results are inconsistent with the conclusions drawn by the systematical review carried out by van Noorden et al. (2015).

The second hypothesis of the study was partially corroborated. Contrary to what was expected, being a girl does not predict a greater likelihood to be victimized in any of the samples from Spain or Ecuador. But it was observed that being a girl predicted a lower involvement in bullying aggressions, which is in line with the conclusion of Kokkinos and Kipritsi’s (2012) study. This is consistent with the fact that female adolescents develop more assertive behaviors than male adolescents in difficult situations among peers (Rose and Rudolph, 2006). An aspect that we should remark in the Ecuadorian sample is that low affective empathy and being a boy are factors that increase the likelihood of being a bully, which is in line with the relation between these aspects as Caravita et al. (2009) stated.

The conclusion is therefore that aggression and victimization among adolescents are predicted reciprocally. This confirms the third initial hypothesis stated following the evidence of the existing overlap between the roles of bully and bullying victim in most of the people involved in this violent phenomenon (e.g., Mishna, 2003; Del Rey et al., 2012).

The results confirm the fourth hypothesis: ethnic-cultural victimization and aggression are predictors of bullying aggression and victimization. The evidence of the important predictive force of involvement in ethnic-cultural victimization over involvement in bullying victimization is a great step forward regarding the observations provided by some previous studies that had already emphasized the existence of some type of relation between both (Rodríguez-Hidalgo et al., 2014, 2015; Cardoso et al., 2017). Victimization can be predicted in terms of involvement in ethnic-cultural aggression. Bullying aggression is predictable depending on involvement in ethnic-cultural victimization and/or ethnic-cultural aggression. From these last findings, no precedents have been found in the scientific literature. It is interesting that ethnic-cultural aggression is a negative predictor of bullying victimization, and ethnic-cultural victimization is a negative predictor of bullying aggression in both samples. As a possible explanation for this, taking into account that the power imbalance between bully and victim plays a key role in the dynamics of bullying (Ortega-Ruiz et al., 2012; Olweus, 2013; Sentse et al., 2017), it is proposed that a significant proportion of this power imbalance between peers in pluricultural contexts is related to the difference of ethnic-cultural status. This ethnic-cultural status could be expressed and built at a large extent between peers depending on the emission and reception of ethnic-cultural discriminatory behaviors. This way, those who attack others discriminatorily could be more feared and less subject to bullying victimization at the same time. Those who suffer discriminatory victimization could be self-perceived and perceived by others with little or no power over their peers, what would confer them a disadvantageous situation to emit aggressive behaviors of bullying toward others.

The development of the research has considered the use of robust and reliable instruments over wide samples in Spain and Ecuador. Educational inferences are set out on the provided conclusions and discussion in order to improve the prevention of bullying, the limitations of the study, and the future lines of research.

A great part of the educational interventions based on the scientific evidence to prevent and palliate bullying has considered the educational work to build up self-esteem and to develop empathy and/or social skills (Ttofi and Farrington, 2011; Boket et al., 2016; Avşar and Ayaz-Alkaya, 2017; Didaskalou et al., 2017; Lösel and Ttofi, 2017). The conclusions of the study contribute to support this line of proposals. Nevertheless, the greatest part of the interventions has disregarded dealing with violent and discriminatory phenomena based on cultural differences (Earnshaw et al., 2018). The study proposes that educational policies and interventions take into account the approach of ethnic-cultural discrimination to prevent bullying, due to its high predictive value on this phenomenon. In the educational centers, the processing of contents and the development of educational strategies are necessary to promote the intercultural coexistence and the eradication of ethnic-cultural discrimination. Recently, new educational proposals against bullying are being developed, paying particular attention to the ethnic-cultural diversity and to the risk of discrimination and exclusion. An example of this is the Educational Model of Intercultural Coexistence—MECI in Spanish—(Rodríguez-Hidalgo et al., 2017). This work proposal is consistent with a budding but emerging line of bullying preventive and palliative psychoeducational models based on the stigma for having one or several socially devalued characteristics (Earnshaw et al., 2018).

Some of the limitations of the study are inherent to the type of methodology and instruments used in the research. As it is a cross-sectional study, the results are aimed toward the prediction and not toward the inference of causal relations between variables. The use of the self-report questionnaire enables us to collect data of self-perception, but not about the hetero-perception among peers. Starting from the results provided about the prediction of bullying aggression and victimization among adolescents, it would be convenient to develop a longitudinal study considering the same variables in the future. This would make possible to know more about the potential causal relations. Additionally, it would be interesting to take measures not only about self-perception but also about hetero-perception among peers. It would be convenient to start a line of studies on bullying and discrimination among adolescents taking into account socioeconomical indicators, as it is being done regarding other violent and/or psychological phenomena in adults (e.g., Mucci et al., 2016; Bartoll et al., 2019). These guidelines would allow us to advance in the study of bullying and ethnic-cultural discrimination as both are ecological and dynamic in the peer network.

## Data Availability

The datasets generated for this study are available on request to the corresponding author.

## Author Contributions

All authors made substantial contribution to the theoretical framework, design, data collection or interpretation of this study. All contributed to this article and approved its publication.

### Conflict of Interest Statement

The authors declare that the research was conducted in the absence of any commercial or financial relationships that could be construed as a potential conflict of interest.
